# Classroom teaching with simulated patients during COVID-19: the communication skills course in the second year of the model medical curriculum HannibaL

**DOI:** 10.3205/zma001374

**Published:** 2020-12-03

**Authors:** Thomas von Lengerke, Kambiz Afshar, Ingo Just, Karin Lange

**Affiliations:** 1 Hannover Medical School (MHH), Department of Medical Psychology, Simulated Patients Programme (SPP-MHH), Hannover, Germany; 2 Hannover Medical School, Institute for General Practice, Module “Diagnostic Methods”, Hannover, Germany; 3 Hannover Medical School, Dean of Medicine, Bachelor and Master Studies, Hannover, Germany

**Keywords:** simulated patients, communication and social skills, doctor-patient communication, medical education, hygiene, COVID-19

## Abstract

**Objective:** In the spring of 2020 in response to the COVID-19 pandemic, the question arose at Hannover Medical School as to how simulated patients (SP) could still be utilized in the communication course that is part of the module “Diagnostic methods” taught in the second year of the model medical curriculum known as HannibaL.

**Methods:** This short report summarizes the process of implementing the utilization of SP in analog classroom teaching and describes the relevant results on the concluding Objective Structured Clinical Examination (OSCE) in comparison to the previous year.

**Results: **Overall, the analog SP deployments were practicable under local conditions and in compliance with precautionary measures to curb the risk of infection, whereby the OSCE scores did not deviate significantly from those in the prior year.

**Conclusion: **During the COVID-19 pandemic and perhaps other epidemics as well, it will continue to be important in the future to make locally adapted, purpose-oriented, and preventively effective decisions regarding university didactics in undergraduate studies.

## Zusammenfassung

**Zielsetzung: **Im Frühjahr 2020 stellte sich angesichts der COVID-19-Pandemie an der Medizinischen Hochschule Hannover die Frage, wie im Gesprächsführungspraktikum des Moduls „Diagnostische Methoden“ im 2. Studienjahr des Modellstudiengangs HannibaL Simulationspersonen (SPs) zum Einsatz kommen können.

**Methodik: **Dieser Kurzbeitrag fasst den Prozess zusammen, der zur Realisierung von SPs-Einsätzen als analoge Präsenzlehre führte, und beschreibt die relevanten Ergebnisse der abschließenden Objective Structured Clinical Examination (OSCE) im Vergleich zum Vorjahr.

**Ergebnisse: **Insgesamt zeigt sich die Realisierbarkeit der analogen SP-Einsätze unter den standortortspezifischen Bedingungen und auf Basis eines adhärenten Hygienekonzepts, wobei die OSCE-Ergebnisse nur unwesentlich von denen des Vorjahres abwichen.

**Schlussfolgerung:** Während der COVID-19-Pandemie und ggf. weiterer Epidemien wird es auch künftig darauf angekommen, unter Berücksichtigung von Standortspezifika hochschuldidaktisch zielführende und präventiv wirksame Entscheidungen zu treffen.

## Introduction

Although there does not appear to be a current scientific definition of “classroom teaching” and, according to media psychologists, a feeling of “presence” can be created in digital teaching [1], it is plausible that there are advantages to analog forms of classroom teaching (in terms of physical presence as well). This holds especially for teaching practical skills, as has been shown by a number of controlled evaluation studies, some of which have been conducted at medical schools [2], [3]. This also applies to communication and social skills – especially when utilizing simulated patients (SP) to impart them. There can be many reasons for doing so:

### Technical

- Software tools (e.g., [4]) are not (yet) available on site;
- Hardware, internet connections and network access are inadequate for SP or students, for instance, in terms of quality or quantity.

#### Educational

- Students are meant to practice communication and social skills for the first time using SP and, for this reason, should experience these interpersonal situations while being physically and psychologically present, doing so with as many senses as possible in a three-dimensional social space;
- The achievement of learning objectives regarding nonverbal behavior or emotions should not be influenced by features of interpersonal situations that are determined by digitalization.

In the 2020 summer semester during the COVID-19 pandemic, the question arose as to how SP could be deployed in the communication course that is part of the module “Diagnostic Methods” taught in the second year of the model medical curriculum known as HannibaL. The aim of the ad hoc interventions described here was to enable students to practice with SP in a classroom setting.

## Project description

The communication course prepares students for two of nine stations (biopsychosocial medical history taking and breaking bad news, each lasting 15 minutes) of an M1-equivalent, cumulative Objective Structured Clinical Examination (OSCE); each of these two stations represents a sixth of the 300 points possible (lowest passing score: 60%; total length of examination: 95 min.; see [5] for details). This course is taken by 28 groups of usually 10 students each and entails seven course sessions (see also [6]). While the first and second sessions that met between February 17 and March 12, 2020, were held face-to-face in the classroom, sessions 3-7 were scheduled to take place between April 20 and June 25, 2020. In an initial response to the pandemic, the format for these sessions was switched to asynchronous online teaching (e-mail sent by the Dean of Studies, April 2, 2020). It was therefore first decided to start the summer trimester with two sessions without SP and to schedule the sessions with SP (medical history taking, breaking bad news, and final exam preparation) as late as possible, meaning on or after May 18, 2020, so as to be in a position to take advantage of any potential easing of restrictions. After outpatient appointments for clinical studies became possible again on May 11, 2020, the university administration also permitted classroom-based teaching with scheduled SP on the condition that the mandatory precautions to prevent infections were complied to (see below). As a result, each student was able to participate as planned in three face-to-face SP appointments.

**Figure 1:**
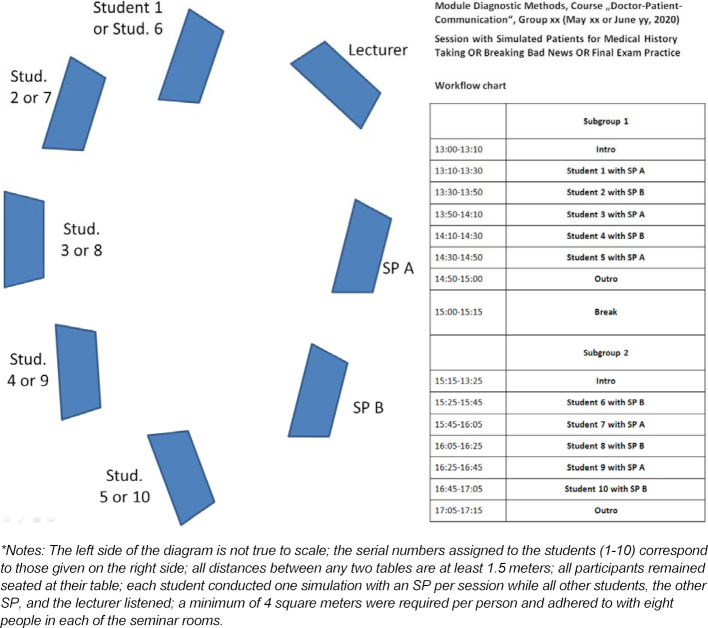
Diagram of the classroom layout (left)* and workflow chart (right) for a communication course session held with SP in an analog setting, module “Diagnostic Methods”, HannibaL model medical curriculum, May 18-June 25, 2020.

Of course, this presumed adherent precautionary measures. In addition to disinfecting hands and surfaces, face masks had to be worn by all participants once they entered the university building until taking their position in the classroom, and while leaving. Figure 1 (Fig. 1) presents a diagram of the classroom layout, in which the prescribed distances were observed at all times. The pre-requisite was dividing the groups, which normally met for three hours per session, into two groups of five each which met for two hours (see the workflow chart in figure 1 (Fig. 1)). The OSCE was scheduled for July 2-10, 2020, in the Skills Lab Hannover (SkilLaH). On June 11, 2020, it was decided that students, examiners, and SP must cover their nose and mouth (face mask) at all exam stations. To best prepare students for the stations that assessed communication skills, a recommendation was made to practice with face masks during the final course session.

Thus, overall a blended-learning approach was pursued, whereby the course sessions remaining after recognition of an epidemic situation of national importance (German Bundestag, March 25, 2020) were originally planned to take place in the form of eLearning, but given the developments described all sessions with SP were conducted in a classroom setting. Comparison with the OSCE scores from the previous year using IBM SPSS® (v26) UNIANOVA and the Mann-Whitney U test for comparing non-normally distributed variables shows no difference in medical history taking (mean=42.1 and median=43.0 in 2020 vs. 41.9 and 43.0 in 2019, p=0.647 and 0.808), breaking bad news (41.7 and 43.0 vs. 41.5 and 43.0, p=0.585 and 0.891), or total points on the OSCE (249.1 and 249.0 vs. 247.8 and 252.0; p=0.459 and 0.268). The percentage of participants who had not registered to take the OSCE was also comparable (2020: 6.6%, 2019: 7.7%; 95% CI for the difference -5.4%|3.2%). Likewise, the evaluation (with 9.0% less participation in 2020) did not deviate in regard to the overall module (12.1 and 12.0 vs. 12.4 and 13.0 points [of 15], p=0.354 and 0.315), SP utilization (1.1 and 1.0 vs. 1.2 and 1.0, p=0.109 and 0.188), or the subjective achievement of the learning objectives for medical history taking (1.3 and 1.0 for each objective, p=0.249 and p=0.263; scale: 1=completely agree to 6=completely disagree). Only the evaluation for breaking bad news was worse, however only statistically and not clinically significant (1.6 and 1.0 vs. 1.4 and 1.0, p=0.024 and 0.054), especially because this did not correspond with the OSCE points (see above) and was also parametrically no longer significant after Bonferroni correction (p<0.0125).

## Conclusion

In light of the pandemic situation during the 2020 summer semester, we do not wish to judge if our approach serves as a model for other universities due to the many site-specific details. We are also aware that the GMA Committee on Simulated Patients has recommended, with reference to Peters et al. [7], foregoing whenever possible direct SP contact in teaching and other educational contexts ([8], p. 1). At the same time, Peters & Thrien [9] do not use the terms “blended learning,” “eLearning,” “digital” or “virtual,” nor did a PubMed search on July 17, 2020, yield relevant results on SP and COVID-19 (algorithm available upon request). Both show, in our opinion, that because of the lack of experience with pandemics, local solutions must be found for using SP in teaching. The fact that even two weeks after the OSCE no SARS-CoV-2 infection became known in connection with the deployment of SP described here shows that, when precautions are taken (physical distancing and face masks), it is possible to have educationally meaningful instruction in classrooms, organized in such a way to minimize the risk of infection. Given the examination and evaluation results, the aim of the ad hoc interventions described here to allow students the opportunity to effectively practice with SP in a classroom setting has evidently been successfully attained.

## Acknowledgements

We thank Dr. Ella Ebadi for her support in developing the hygienic concept for our specific classroom setting, Zada Akyol for her tireless administrative support, and of course all simulated patients for participating in such a challenging time.

## Competing interests

The authors declare that they have no competing interests.
